# Predictors of non-adherence to antiretroviral therapy among HIV infected patients in northern Tanzania

**DOI:** 10.1371/journal.pone.0189460

**Published:** 2017-12-18

**Authors:** Seleman Khamis Semvua, Catherine Orrell, Blandina Theophil Mmbaga, Hadija Hamis Semvua, John A. Bartlett, Andrew A. Boulle

**Affiliations:** 1 Kilimanjaro Christian Medical Centre-Duke Research Collaboration, Moshi, Tanzania; 2 Desmond Tutu HIV Centre, University Of Cape Town, Cape Town, South Africa; 3 Kilimanjaro Christian Medical University College, Moshi, Tanzania; 4 Duke Global Health Institute, Durham, North Carolina, United States of America; 5 Kilimanjaro Clinical Research Institute-Kilimanjaro Christian Medical Centre, Moshi, Tanzania; International AIDS Vaccine Initiative, UNITED STATES

## Abstract

**Background:**

Antiretroviral therapy (ART) has been shown to reduce HIV-related morbidity and mortality amongst those living with HIV and reduce transmission of the virus to those who are yet to be infected. However, these outcomes depend on maximum ART adherence, and HIV programs around the world make efforts to ensure optimal adherence. Predictors of ART non-adherence vary considerably across populations and settings with respect to demographic, psychological, behavioral and economic factors. The objective of this study is to investigate risk factors that predict non-adherence to antiretroviral treatment among HIV-infected individuals in northern Tanzania.

**Methods:**

At Kilimanjaro Christian Medical Centre (KCMC), a tertiary and referral hospital in northern Tanzania, we used an existing ART database to randomly select HIV-infected patients above 18 years of age who have been on triple ART for at least two years. We used interviewer administered structured questionnaires to cross-sectionally determine predictors of ART non-adherence. We determined non-adherence through retrospective review of pharmacy drug refill (PDR) records of the interviewed participants using a pharmacy database.

**Results:**

Non-adherence was defined as collecting less than 95% of expected monthly refills in the previous 2 years. Multivariable logistic regression model was used to determine the predictors of non-adherence. Of the 256 patients enrolled mean age was 44 years (SD ± 11) and median CD4 count was 499 cells per microliter (IQR 332–690). Median PDR adherence was 71% (IQR 58%–75%). Non-adherence was associated with younger age and unemployment.

**Conclusion:**

In this setting, adherence strategies could be adapted to address issues facing young adults, and those with household challenges such as unemployment. Further research is required to better understand the potential roles of these factors in suboptimal adherence.

## Introduction

The use of antiretroviral therapy (ART) has been shown to prolong and improve the quality of life of people living with HIV/AIDS[1] and reduce transmission of the virus to those who are yet to be infected[2] In the past decades the World Health Organization (WHO) has been focusing on scaling up ART coverage[3, 4] and as of 2015, 17 million people living with HIV globally were on antiretroviral medication[5] In Tanzania 1.4 million people were living with HIV and 53% of them were already on antiretroviral treatment by 2015[6]after the government’s efforts to strengthen and scale up free and comprehensive care and treatment services in public and private facilities[7]

Successful antiretroviral therapy results in viral suppression, immunologic improvement, and reduced viral transmission as well as reduced drug resistance. These outcomes depend substantially on consistent adherence to treatment[8, 9]. Data from prospective studies associate suboptimal adherence with drug resistance[9], reduced viral suppression and immunologic failure[8] as well as progression to AIDS[10]. However maintaining optimal adherence and retention of patients in care and treatment programs has been a major challenge[1], and therefore increased efforts to promote adherence are important for successful antiretroviral therapy.

Non-adherence to antiretroviral therapy has been associated with a range of factors including sociodemographic, psychological, socioeconomic, sociobehavioral and contextual factors[11–13]. Although ART non-adherence is influenced by these factors, a systematic review of studies done in developed and developing nations suggest that the barriers to adherence are consistent across settings and countries[14]. Another systematic review and meta-analysis of studies done in low, middle and high income countries shows a higher proportion of people living with HIV (PLHIV) in low income countries who achieve good adherence as compared to PLHIV in high income countries[15]. Studies suggest that most of the barriers to optimal adherence are consistent in both developed and developing settings, although there are contextual barriers such as issues of access which are more common in developing countries[14]. Nevertheless, optimal levels of ART adherence can be achieved in sub Saharan African settings if barriers to optimal adherence are addressed[16, 17].

Since the gold standard approach for assessing ART adherence has not been established [18, 19] many researchers use patient self-reporting because of its low cost and simplicity[20], although this measure has led to underestimation of non-adherence[21]. Pharmacy adherence measures (PAM) are also relatively simple to administer and have provided more clinically relevant results compared to self-reports[22–24].

Adherence is a dynamic process that changes over time[25] and predictors of non-adherence vary considerably and therefore no single factor has been consistently associated with non-adherence across all studies[26, 27]. This underscores the need to conduct studies to determine potential predictors of non-adherence in different contexts. Whereas cross sectional and qualitative studies suggest a number of potential predictors of ART non-adherence in northern Tanzania after initiation of free HIV care and treatment, and suggest programs to improve adherence[28, 29], the current prevalence of these predictors has not been quantified. In this study we sought to quantify non-adherence among northern Tanzanian HIV-infected patients on ART for at least two years, measure the potential predictors of non-adherence and explore associations with available adherence measures. The findings of this study will inform contextually tailored adherence interventions.

## Materials and methods

### Study setting

This study was conducted at Kilimanjaro Christian Medical Center (KCMC) in the northern part of Tanzania. The hospital is a non-profit organization under the Good Samaritan Foundation (GSF) of the ELCT, working in memorandum of understanding with the Tanzania government. It is a tertiary referral hospital which serves in-patient and out-patients referred or directly received from four regions (Kilimanjaro, Arusha, Tanga and Manyara) which form the northern part of Tanzania. The hospital HIV care and treatment department registers admitted patients diagnosed with HIV infection in the wards and out-patients diagnosed in the HIV counseling and testing unit. The HIV care and treatment department is divided into two parts, (a) HIV care and treatment clinic (CTC) which takes care of HIV infected adult individuals (b) child centered and family care clinic (CCFCC) which takes care of children and their families.

Based on the current (2012) local HIV care guidelines[30], HIV-infected patients with CD4 counts below 350 cells/mm^3^ regardless of the WHO clinical stage or with WHO stage 3 or 4 regardless of CD4 counts are eligible for antiretroviral therapy. Counseling for HIV-infected patients is done by trained nurses before and after treatment initiation. Based on clinical status and baseline laboratory tests, clinic physicians initiate medications while clinic pharmacists are responsible for final counseling and dispensing. Continued drug refill visits are scheduled every one or two months depending on patient’s clinical status and hospital ART stock status. The hospital HIV care and treatment team is responsible for assessing patient progress as they visit for drug refills. The clinic pharmacy also stocks drugs for opportunistic infections for when these are prescribed.

### Inclusion and exclusion

The study included HIV-infected patients ≥18 years of age, who had been on triple antiretroviral drugs as part of their HIV lifelong care for at least two years and who could provide informed consent. The study excluded patients with impaired recall capacity based on interviewer assessment, and those who are in the KCMC ART database as occasional visitors but not primarily enrolled as KCMC HIV care and treatment clinic patients.

### Study design, sampling and recruitment

A cross-sectional survey design was used. The target sample size was 214 eligible HIV infected patients calculated to offer 80% power to detect a 14% non-adherence among men as compared to women, the two groups equally distributed in the study population[29, 31, 32]. A simple random sampling was done using the clinic database. A random sample of 257 (214 + 20% to account for losses) out of 2315 patient identification numbers was selected from the clinic database. Initial screening was done based on age and duration on ART. Eligible patient’s clinic visit dates were identified using the appointment register. These patients were contacted through mobile phone calls and/or physically by the interviewers on the day of their clinic visit. The interviewers requested private meetings (after completion of clinic visit) with patients and introduced the study. For patients who had enough time, the oral consenting process started immediately and written consent forms for further reading were provided. For those who did not have enough time on the day, a meeting on a different day was requested at the patient’s convenience. Once the patient was ready to provide written informed consent interviews were planned and conducted.

### Measurements

Pharmacy drug refills (PDR) were reviewed for the two years preceding the interview to enable a calculation of adherence based on retrospective PDR data. Adherence was measured based on the number of refills done out of 24 refills expected in the past two years. Less than 95% PDR was considered non-adherence as defined by Tanzania Ministry of Health. Participants were also asked to recall their pill-taking history over the preceding four days for a second measure of adherence. Predictors of and reasons for ART non-adherence were elicited using closed and open-ended questions in a structured questionnaire that was adapted from ACTG validated questionnaire[33]. In addition to the study interest in longer term adherence, the inclusion of patients with at least two years of follow up facilitated the first adherence measure (Pharmacy Adherence Measure) used in this study. A total of four months (from mid-July to late November 2015) were used for data collection, three months of participant interviews and one month of reviewing PDR records.

Demographic information included age, gender, marital status, level of education, occupation and residence (rural versus urban). Recall non-adherence was assessed by calculating the proportion of doses missed in the previous four days as recalled by patients. The last time ART medication was missed was assessed and categorized as never, >3 months ago, 1–3 months ago, 2–4 weeks ago, 1–2 weeks ago and within the past week. The past 4-days adherence was also assessed by asking “During the past 4 days how many days have you missed taking all your doses?” Behavioral reasons for non-adherence were elicited by listing the predetermined factors and asking participants “How often do the following reasons make you miss your medication?” The response was never, sometimes or often. An open-ended question was formulated to enable participants list other factors not covered in the list. Patient satisfaction with the service offered to them by clinicians, nurses, pharmacists and laboratory personnel was assessed through a “yes” or “no" response to a question enquiring about their satisfaction. Participant awareness of the current line of treatment, baseline and current CD4 counts was measured by asking “What line of treatment are you currently on?” and “What was your baseline CD4 count/What is your current CD4 count?” The response was cross-checked with the treatment regimen and CD4 count information recorded on the participants’ CTC cards.

## Non-adherence

In this study non-adherence was defined as less than 95% of the expected 24 monthly pharmacy drug refills (PDR) of two years past the pharmacy retrospective data review. A complementary threshold was used for sensitivity analysis to determine whether the same risk factors will be found based on 80% PDR. The 95% threshold is consistent with the Tanzania Ministry of Health and WHO recommendation for ART adherence[1, 34]. Patients 4-days self-recall was also used as a second measure to determine non-adherence at 95% threshold by calculating the percentage number of pills taken in the past 4 days.

## Statistical analysis

For the descriptive analysis of the patient characteristics, mean and standard deviations (SD) were calculated for normally distributed variables whereas median and interquartile ranges (IQR) were calculated for skewed variables. An adherence threshold of 95% and a complementary threshold of 80% were used to report non-adherence based on both adherence measures. Adherence levels of interviewed participants (using the two thresholds) were compared based on the sociodemographic characteristics. Adherence levels were also compared across most recent CD4 count strata and by duration on ART. Chi-squared p-values were calculated for each comparison. Assessments of associations with ART non-adherence were done based on an a priori multivariable logistic regression model. This method was preferred since the interest was to test the risk factors to ART non-adherence as determine by prior studies. Age, sex, employment, education, marital status, residences, duration on ART and most recent CD4 count were considered to be the potential predictors of non-adherence based on literature. They were all included in the multivariable model and their association with non-adherence was ascertained. The reasons for non-adherence were assessed by calculating the proportion of patients who reported each reason among all included participants, and also by calculating this proportion among participants who were found to be non-adherent at the 80% threshold based on PDR measure. Measures of effect were interpreted based on the size of effect and p-value, without reliance on a dichotomous cut-off of significance which would have artificially differentiated findings with broadly similar statistical support.

## ART program in Tanzania

The ART program changes with time as research reveals new HIV/AIDS related information and the WHO point out new recommendation regarding management of HIV/AIDS. At the period of this research the program was using the Tanzania ministry of Health guideline 2012. The PDR data collection and analysis was done immediately after the recruitment and interviews and no significant changes occurred in the program

## Ethical clearances

Ethical clearance approvals were secured from the Kilimanjaro Christian Medical University College (KCMUCo) and University of Cape Town (UCT) ethics committees. Oral and written consent were obtained from each patient before enrollment into the study. Interviews were done in special clinic rooms, and patient ID numbers were used and names were kept anonymous.

## Results

**Fig 1:**
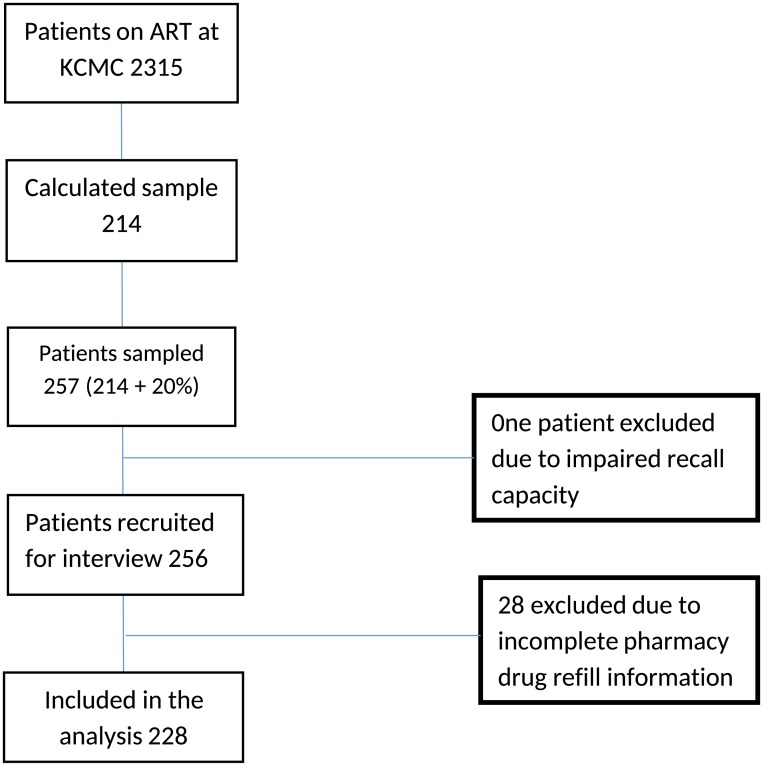
Patient flow schema.

A total of 257 patients were recruited to participate in the study. One participant was excluded due to impaired recall capacity as a result of a mental health problem and 28 participants who were not different from the overall sample (Table 1) were excluded due to incomplete prescription refill information, and therefore a total of 228 patients were included in the regression analysis. Fig 1 shows numbers of patients included in each step of the study from recruitment for interview to analysis. Approximately two thirds (66.2%) of the participants were female. The mean age of the participants was 44 years (SD: ± 11) and the median duration on ART was 4.5 years (IQR: 3–6.8). Median PDR adherence was 71% (IQR 58%–75%). The median CD4 count at the point of ART initiation was 105 cells per microliter (IQR: 49–201) and the median CD4 count at the point of interview was 499 cells per microliter (IQR: 332–690). Just over half of participants were currently married (51.3%). 57.0% reported residing in a township and 21.5% were not employed (Table 2).

**Table 1:** Demographic characteristics of the recruited and excluded patients.

Category	Included participants N-256%Proportion (95%CI)/ Mean (standard deviation)	Excluded participants N-28%Proportion (95%CI)/ Mean (standard deviation)	P Values
Age (years)	Mean 43.6 (SD 11.0)	Mean 42.8 (11.6)	
Male	32% (95%CI 26–37)	Mean 14% (95%CI 13–27)	0.057
Marital status			
• Never married	23% (95%CI 18–28)	18% (95% CI 4–32)	0.562
• Currently married	50% (95%CI 44–57)	43% (95%CI 25–61)	0.449
Education level			
• Secondary education	25% (95%CI 20–31)	29% (95%CI 12–45)	0.715
• Primary education	61% (95%CI 55–67)	61% (95%CI 42–79)	0.986
Occupation			
• Employed	77% (95%CI 72–82)	64% (95%CI 47–82)	0.141
Residence			
• Urban	56% (95%CI 50–62)	46% (95%CI 28–65)	0.330
Duration on ART (years)	Median 4.5 (IQR 3.6)	Median 3 (IQR 2)	

**Table 2:** Demographic characteristics of the participants (N = 228).

Sociodemographic Characteristics	Number	Percentage
Age (years)		
• < = 29	21	9.2
• 30–39	45	19.7
• 40–49	82	36.0
• 50–59	45	19.7
• 60 and above	18	7.9
• Could not recall birth date	7	3.1
• Mean age 44 years (SD: 11years)		
Gender		
• Males	77	33.8
• Females	151	66.2
Marital status		
• Never married	53	23.3
• Currently married	117	51.3
• Separated/Divorced/Widowed	58	25.4
Education level		
• No education	1	0.4
• Primary education	138	60.5
• Secondary education	57	25.0
• College/University	32	14.1
Occupation		
• Unemployed	49	21.5
• Employed	55	24.1
• Self employed	123	54.0
• Occupation not reported	1	0.4
Current CD4 counts (cells per microliter)		
• < 200	14	06.1
• 200–<350	35	15.4
• 350–<500	41	18.0
• 500–<750	55	24.1
• > = 750	83	36.4
• Median CD4 count 449 cells/μL (IQR 332–690)		
Residence		
• Urban	130	57.0
• Rural	97	42.5
• Residence not reported	1	0.4
Duration on study (years)		
• 2–<4	93	40.8
• 4–<6	51	22.4
• 6–<8	40	17.5
• 8–<10	26	11.4
• > = 10	18	7.9
• Median duration 4.5 years (IQR: 3–6.8)		

## Participants’ non-adherence

Median adherence for PDR was 71% (IQR: 58%–75%) and for patient-recall was 100%. At 95% adherence threshold, 42% (95%CI: 36–49%) and 10% (95%CI: 5–11%) of the participants were non-adherent based on PDR and patient-recall measures, respectively. At the 80% adherence threshold, 25% (95%CI: 20–31%) and 7% (95%CI: 6–14%) of the participants were non-adherent based on PDR and patient-recall measures, respectively.

## Pharmacy drug refill pattern of adherence based on participant characteristics

Table 3 shows that, 78% of participants older than 40 years of age and 68% of those ≤ 40 years of age were adherent at 80% adherence threshold (p = 0.076). The highest proportion of adherent participants based on education levels was amongst those with college/university education (91%) followed by those with primary education (73%) and those with secondary education (70%., p = 0.046). 78% of employed participants as well as those who are self-employed were adherent while only 61% of those who are unemployed were adherent at 80% threshold (p = 0.057). The same trend was observed when the threshold of 95% was used: 62% of employed and 63% of self-employed was adherent while only 39% of unemployed participants were adherent (p = 0.010). The proportion of adherent participants varied across CD4 groups with lowest adherence in those with current CD4 cell counts of 350 to 500 cells/ μL. Adherence was also lowest among patients with longest (> = 10 years) duration on ART.

**Table 3:** Adherence of interviewed participants based on demographic characteristics.

Category	Adherent (0.80 threshold) N-228	P values	Adherent (0.95 threshold) N-228	P Values
Age (years)				
• < = 40	57 (68%)	0.076	43 (51%)	0.117
• > 40	113 (78%)		89 (62%)	
Sex				
• Females	112 (74%)	0.850	86 (57%)	0.687
• Males	58 (75%)		46 (60%)	
Marital status				
• Never married	39 (74%)	0.261	31 (58%)	0.720
• Currently married	92 (79%)		70 (60%)	
• Divorced/Separated/Widowed	39 (67%)		31 (53%)	
Education level				
• No education	0 (0.0%)	0.046	0 (0%)	0.125
• Primary education	101 (73%)		76 (55%)	
• Secondary education	40 (70%)		32 (56%)	
• College/University	29 (91%)		24 (75%)	
Occupation				
• Unemployed	30 (61%)	0.057	19 (39%)	0.010
• Employed	43 (78%)		34 (62%)	
• Self employed	96 (78%)		78 (63%)	
Current CD4 counts (cells per microliter)				
• < 200	12 (86%)	0.050	8 (57%)	0.526
• 200–<350	29 (83%)		24 (69%)	
• 350–<500	24 (59%)		20 (49%)	
• 500–<750	45 (82%)		33 (60)	
• > = 750	60 (72%)		47 (57%)	
Residence				
• Urban	94 (72%)	0.392	78 (60%)	0.419
• Rural	75 (77%)		53 (55%)	
Duration on ART (years)				
• 2–<4	73 (78%)	0.016	62 (67%)	0.119
• 4–<6	37 (73%)		26 (51%)	
• 6–<8	34 (85%)		24 (60%)	
• 8–<10	18 (69%)		13 (50%)	
• > = 10	8 (44%)		7 (39%)	

## Multivariable analysis of the predictors of pharmacy drug defill non-adherence based on 95% adherence threshold

The final multivariable model (Table 4) suggested that, non-adherence to ART was associated with younger age, with an adjusted OR for every 10-year increase in age of 0.54 (95% CI: 0.36–0.80, p = 0.002), Non-adherence was also associated with unemployment, adjusted OR 2.89 (95% CI: 1.21–6.86, p = 0.016).

**Table 4:** Unadjusted and adjusted odds ratios (OR) for predictors of non-adherence to antiretroviral therapy among HIV-infected north Tanzanians (95% adherence threshold).

Sociodemographic variables	Unadjusted OR (95% CI)	P -values	Adjusted OR (95% CI)	P-values
Age				
• For every 10 years increase	0.75 (0.59–0.96)	0.025	0.68 (0.49–0.93)	0.017
Gender				
• Females	REF	0.687	REF	0.338
• Males	0.89 (0.51–1.56)		1.43 (0.69–2.96)	
Marital status				
• Never married	REF	0.721	REF	0.417
• Currently married	0.95 (0.49–1.83)		1.38 (0.62–3.07)	
• Divorced/separated/widowed	1.23 (0.58–2.60)		2.20 (0.85–5.67)	
Education level				
• College/University	REF	0.093	REF	0.080
• Secondary education	2.34 (0.90–6.10)		2.00 (0.71–5.68)	
• Primary education	2.49 (1.04–5.92)		1.95 (0.76–5.02)	
Occupation				
• Employed	REF	0.003	REF	0.008
• Unemployed	2.68 (1.40–5.13)		2.73 (1.30–5.73)	
Residence				
• Rural	REF	0.417	REF	0.939
• Urban	0.80 (0.47–1.37)		0.98 (0.52–1.83)	
Duration on ART				
• Every 1yr (12months) increase	1.08 (0.96–122)	0.184	1.11 (0.98–1.27)	0.113
CD4 counts (most recent)				
• < 350 cells/μL	REF	0.024	REF	0.401
• 350–< 500 cells/μL	1.98 (0.85–4.62)		1.93 (0.75–5.09)	
• 500–< 750 cells/μL	1.25 (0.56–2.79)		1.09 (0.43–2.75)	
• > = 750 cells/μL	2.00 (0.81–4.92)		1.93 (0.66–5.63)	
• Unrecorded	1.15 (0.51–2.62)		1.33 (0.52–3.39)	

## Multivariable analysis of the predictors of pharmacy drug refill non-adherence based on 80% adherence threshold

The final multivariable model (Table 5) suggested that, non-adherence to ART was associated with younger age, with an adjusted OR for every 10-year increase in age of 0.54 (95% CI: 0.36–0.80, p = 0.002), and marital status with adjusted OR for being previously married of 4.15 compared to never being married (95% CI: 1.29–13.29, p = 0.024 across all categories). Non-adherence was also associated with unemployment, adjusted OR 2.89 (95% CI: 1.21–6.86, p = 0.016), and staying in urban areas with an adjusted OR of 2.25 (95% CI: 1.02–4.98, p = 0.045). While there was statistical support for an association between higher most recent CD4 counts and non-adherence, this was not consistent across all levels of CD4 count when compared to those below 350 cells/μl. There was weaker statistical support for the association between ART non-adherence and education level with adjusted OR for primary education level compared to tertiary education of 4.03 (95% CI: 1.26–12.84, p = 0.116 across all categories, (Table 3), as well for male gender, with the OR for men being 2.07 (95% CI 0.86–4.99, p = 0.106)

**Table 5:** Unadjusted and adjusted odds ratios (OR) for predictors of non-adherence to antiretroviral therapy among HIV-infected north Tanzanians using 80% adherence threshold.

Sociodemographic variables	Unadjusted OR (95% CI)	P -values	Adjusted OR (95% CI)	P-values
Age				
• For every 10 years increase	0.67 (0.51–0.90)	0.007	0.54 (0.36–0.80)	0.002
Gender				
• Females	REF	0.850	REF	0.106
• Males	0.94 (0.50–1.77)		2.07 (0.86–4.99)	
Marital status				
• Never married	REF	0.267	REF	0.024
• Currently married	0.76 (0.36–1.61)		1.26 (0.48–3.31)	
• Divorced/separated/widowed	1.36 (0.60–3.08)		4.15 (1.29–13.29)	
Education level				
• College/University	REF	0.046	REF	0.116
• Secondary education	4.11 (1.10–15.34)		3.64 (0.84–15.72)	
• Primary education	3.64 (1.05–12.64)		4.03 (1.26–12.84)	
Occupation				
• Employed	REF	0.018	REF	0.016
• Unemployed	2.26 (1.15–4.44)		2.89 (1.21–6.86)	
Residence				
• Rural	REF	0.392	REF	0.045
• Urban	1.31 (0.71–2.41)		2.25 (1.02–4.98)	
Duration on ART				
• Every 1 yr (12months) increase	1.01 (0.93–122)	0.352	1.11 (0.95–1.30)	0.175
CD4 counts (most recent)				
• < 350 cells/μL	REF	0.024	REF	0.007
• 350–< 500 cells/μL	3.63 (1.36–9.67)		5.03 (1.52–16.63)	
• 500–< 750 cells/μL	1.14 (0.41–3.16)		0.87 (0.25–3.00)	
• > = 750 cells/μL	2.93 (1.04–8.27)		3.41 (0.93–12.53)	
• Unrecorded	1.46 (0.53–3.97)		2.25 (0.66–7.61)	

## Prevalence of patient self-reported reasons for missing ART pills in three months preceding the interview

Table 6 describes the prevalence (in the overall sample and among those who were PDR non-adherent at 80% threshold) of different reasons that were attributing to missing ART pills in the three months preceding the interview. In the overall sample most prevalent factors were traveling away from home (21.5%), forgetfulness (34.2%), running out of pills (15.4%) and busy working for survival (15.8%). Other reasons which were reported in the overall sample by at least 5% of patients included avoiding side effects (6.6%), feeling healthy (7.0%), taking alcohol (6.6%), failing to meet dietary instructions (5.7%), transport problems to the clinic (6.1%), events perceived to be related to taking ART (5.3%), getting tired of too many pills (5.3%), fear of stigmatization by people outside family (7.5%), fear of stigmatization by family members (7.0%), waking up too early for work (11.4%) and financial constraints (8.3%). Reasons which were more prevalent in a subsample of participants who were PDR non-adherent included, avoiding side effect (10.3%) feeling healthy (12.1%), taking alcohol (12.1%), religious belief (10.3%), not fully understanding regimen (6.9%), travelling away (29.3%), having too many pills to take (6.9%) and being tired of taking too many pills (10.3%).

**Table 6:** Patient self-reported reasons for missing ART pills three months preceding the interview.

	Reason attributed to missing ART pills	Proportion of those who reported	Proportion of those who reported among the 80% PR non-adherent
1	Avoiding Side effect	15 (6.6%)	6 (10.3%)
2	Feeling health is okay	16 (7.0%)	7 (12.1%)
3	Took alcohol	15 (6.6%)	7 (12.1%)
4	Could not meet dietary instructions	13 (5.7%)	1 (1.7%)
5	Sharing drugs with other family members and friends	2 (0.9%)	1 (1.7%)
6	Religious belief	11 (4.8%)	6 (10.3%)
7	Not fully understanding regimen and its requirements	8 (3.5%)	4 (6.9%)
8	Travelled away from home	49 (21.5%)	17 (29.3%)
9	Transportation problem getting to clinic	14 (6.1%)	1 (1.7%)
10	Lost pills	2 (0.9%)	1 (1.7%)
11	Had too many pills to take	11 (4.8%)	4 (6.9%)
12	Bad events thought to be related to taking pills happen	12 (5.3%)	4 (6.9%)
13	Forgot	78 (34.2%)	21 (36.2%)
14	Ran out of pills	35 (15.4%)	9 (15.5%)
15	Tired of taking too many pills	12 (5.3%)	6 (10.3%)
16	Busy doing other things. Working for survival	36 (15.8%)	6 (10.3%)
17	Other illness or health problems	10 (4.4%)	2 (3.5%)
18	Fear of stigmatization by people outside the family	17 (7.5%)	4 (6.9%)
19	Fear of stigmatization within family members (not wanting husband/wife/kids/parents to know)	16 (7.0%)	4 (6.9%)
20	Pills got damaged by heat or water	0 (0.0%)	0 (0.0%)
21	Too ill to attend clinic for drugs refill	5 (2.2%)	1 (1.7%)
22	Waking up too early for work. No time to eat	26 (11.4%)	5 (8.6%)
23	Don’t think drugs really work	8 (3.5%)	3 (5.2%)
24	Financial constraints. Had no money for bus fair to clinic	19 (8.3%)	3 (5.2%)
25	Taking substance. Dagga, drugs etc	0 (0.0%)	0 (0.0%)

## Discussion

### Principal findings

In this setting, 42% of individuals on ART collected under 95% of their pharmacy drug refills in the two years prior to the interview, and 25% were considered non-adherent using collection of less than 80% of their PDR as the adherence threshold. Patient recall non-adherence was lower and poorly correlated with PDR non-adherence, 10% and 7% at <95% and <80% thresholds, respectively. Factors with some evidence of association with non-adherence were younger age and being unemployed in both adherence thresholds, while the use of a more lineant threshold of 80% showed additional association with marital partnership characteristics, low education level, higher CD4 counts, male gender and living in urban areas[35, 36].

### Comparison with other studies

Previous studies done both in this setting[29, 37] and other resource-limited settings[38, 39], have demonstrated low levels of ART non-adherence consistent with the patient recall non-adherence data in this study. A meta-analysis of studies which used both of the approaches used in this study and measured non-adherence at different thresholds, reported overall non-adherence as high as 32% among African ART users[16]. This level of non-adherence, which is likely to have been underestimated as the majority of included studies used self-reported measures to estimate adherence, is consistent with the PDR non-adherence in our study.

Younger age has been associated with non-adherence among Tanzanian ART users[32, 40] as in other African studies[41, 42]. We saw a similar trend in our study. This is of concern as the majority of HIV infections occur in this age group[43–46]. Some studies have suggested increased non-adherence among men[32, 35, 36, 47, 48]. While this was also the case in our study, the there was only modest statistical support for this association. There is varying data on the impact of relationship status with non-adherence. Some studies have not found an association between relationship status and non-adherence while others have found marriage to be risk factor[38] for poor adherence. In our setting there was an association between poor adherence and marriage ending either due to dissolution or death. There was also a positive association between education and adherence levels in our study similar to Yaya et al’s observation in Togo, a typical Sub-Saharan African context[49].

Review of literature suggests that, in low and high (but not middle) income countries, employment status of HIV-infected patients impacts ART adherence[41, 50]. Our study found that, ART non-adherence in the Tanzanian setting is associated with unemployment. Similar to other studies, for example, Muya et al[32], which associated non-adherence with increased CD4 count and duration on ART, this study found increased risk for ART non-adherence with higher CD4 counts, although the association was inconsistent across CD4 count strata and warrants further exploration. There was also an association between ART non-adherence and living in urban areas as it was observed in the study of veteran ART users in the United States[51].

### Strengths and weaknesses of the study

This study adapted a validated questionnaire to elicit potential predictors of ART non-adherence and to determine 4-day adherence recall[33]. Unlike most other studies, we determined non-adherence using two measures (PDR and patient recall) to provide a dual assessment of adherence. Although the PDR method assumes complete and accurate data entry as well as complete swallowing of the collected pills, it still provides an effective and simple way of measuring non-adherence[22] and has been observed to offer clinically relevant information[23, 41]. Patient recall measures are well known for subjectivity, recall bias and underestimation of non-adherence[20, 21, 52, 53] nonetheless the correlation with viral load, affordability and simplicity [20, 54, 55] cannot be overlooked. We argue that, incomplete data entry which underestimates adherence may have attributed to profound difference in non-adherence-estimates of the two measures. The large difference in non-adherence estimates might also have been the result of the sampling approaches, one being cross-sectional and the other integrating data over a 2-year period. Since the two measures were poorly correlated in our study, we preferred using PDR over the patient recall measure in further analyses based on findings and recommendations from previous studies[22–24], and therefore in addition to the strength of using two adherence measures, associations were assessed against the more robust measure. However, this study suffers the limitation of under-assessment of adherence due to possible incorrect and incomplete data entries. The study was also under-powered for testing many of the associations due to the limited sample size. The chosen approach is informative, although does not use longitudinal analysis techniques to analyze longitudinal data and therefore ignores the complex nature of dealing with complicated drug prescription data.

### Implications of the study findings and future research

These results highlight that, in order to achieve minimal levels of non-adherence, the ART care and treatment team should focus adherence interventions in patients who are younger, those who were previously married and those with lower levels of education. Adherence intervention could also be focused on patients who are not employed, and possibly men, those with high CD4 counts and those who live in urban areas. Further, perhaps qualitative, research could be used to gain a better understanding of youth-specific ART concerns and to identify manageable issues for those who are unemployed or have lost the support of marital partners, to inform future interventions. Achieving high levels of adherence will improve virological, immunological and clinical outcomes and offer better quality and prolonged life.

### Conclusion

The study has demonstrated that a substantial proportion of patients are sub-optimally adherent to ART, and that there are a number of identifiable associations with non-adherence which could guide future interventions to improve adherence support. Adherence promotion strategies could be adapted to address youth-specific factors, and household factors such as loss of support from a spouse and unemployment. Additional patient groups in whom further exploration of adherence issues is warranted include men, those in urban settings and potentially those with higher recent CD4 counts.

## Supporting information

S1 Data Semvua-dataxx. (XLS)

S1 Questionnaire Questionnaire—Predictors of non-adherence. (DOCX)
