# Supplementary material for: Predictors of non-adherence to antiretroviral therapy among HIV infected patients in northern Tanzania
Source: PLoS One. 2017 Dec 18;12(12):e0189460. doi: 10.1371/journal.pone.0189460 (PMC5734684; doi:10.1371/journal.pone.0189460)
Supplement: S1 Questionnaire — (DOCX) [file pone.0189460.s002.docx]

**Study questionnaire (English)**

**SECTION A: DEMOGRAPHIC INFORMATION**

Tick in the box

1. Sex

Male Female

1. Birthday ……………………………….
2. Marital status
3. Single b) Married c) Divorced/Separated/Widowed
4. Level of education? Please tick in the box.
5. No education
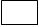


1. Primary education

1. Secondary education

1. College/University
2. Occupation
3. Peasant/Farmer
4. Employed

1. Un-employed

1. Business

1. Self-employed
2. Residence

| Moshi | Town |  |
| --- | --- | --- |
|  | Rural |  |
| Outside Moshi | Town |  |
|  | Rural |  |

**SECTION B; DRUG ADHERENCE ASSESSMENT**

1. How many doses did you miss? *Fill the below table using patient self-recall responses*.

- ***If no dose was missed write zero in the box***

***- The question asks about doses not pills. If only a portion of the dose was taken report the dose as being missed***

| Drug code | Drug name/abbreviation | Number of prescribed doses per day | Number of prescribed doses missed yesterday | Number of prescribed doses missed day before yesterday (2days ago) | Number of prescribed doses missed 3 days ago | Number of prescribed doses missed past 2 weeks |
| --- | --- | --- | --- | --- | --- | --- |
|  |  |  |  |  |  |  |
|  |  |  |  |  |  |  |
|  |  |  |  |  |  |  |

***Past 2 weeks include the doses missed yesterday, 2 days ago and 3 days ago***

1. When was the last time you missed taking your antiretroviral medication?

| 0 | Never |  |
| --- | --- | --- |
| 1 | More than three months ago |  |
| 2 | 1-3 months ago |  |
| 3 | 2-4 weeks ago |  |
| 4 | 1-2 weeks ago |  |
| 5 | Within the past weeks |  |

1. During the past 4 days how many days have you missed taking all your doses.

| 0 | Never |  |
| --- | --- | --- |
| 1 | One day |  |
| 2 | Two days |  |
| 3 | Three days |  |
| 4 | Four days |  |

**SECTION C: REASONS FOR NON-ADHERENCE**

1. Various reasons attribute to people missing taking their medication. Below is a list of possible reasons that may have made you miss your medication in the past month

How often do the following reasons made you miss your medications?

|  |  | 0.Never | 1.Sometimes | 2.Often |
| --- | --- | --- | --- | --- |
| 1 | Wanted to avoid side effects |  |  |  |
| 2 | Felt my health is ok. |  |  |  |
| 3 | I took alcoholic drinks |  |  |  |
| 4 | Could not follow dietary instructions |  |  |  |
| 5 | Sharing drugs with other family members and friends |  |  |  |
| 6 | Religious beliefs |  |  |  |
| 7 | Not fully understanding the regimen and its requirements |  |  |  |
| 8 | I travelled away from home |  |  |  |
| 9 | Transportation problems getting to the clinic |  |  |  |
| 10 | Lost pills |  |  |  |
| 11 | Had too many pills to take |  |  |  |
| 12 | Bad event I thought was related to taking pills happened |  |  |  |
| 13 | Forgot |  |  |  |
| 14 | Ran out of pills |  |  |  |
| 15 | Tired of taking too many pills |  |  |  |
| 16 | Busy doing other things. Working for survival |  |  |  |
| 17 | Other illness or health problems |  |  |  |
| 18 | Fear of stigmatization by people outside the family |  |  |  |
| 19 | Fear of stigmatization within family members (not wanting husband/wife/kid/parents to know) |  |  |  |
| 20 | Pills got damaged by heat or water |  |  |  |
| 21 | Too ill to attend clinic for drugs refill |  |  |  |
| 22 | Waking up too early for work. No time to eat |  |  |  |
| 23 | Don’t think they really work |  |  |  |
| 24 | Financial constraints. Had no money for bus fair to clinic |  |  |  |
| 25 | Taking substances. Dagga, drugs etc |  |  |  |

1. Others reason(s) not listed 26.

27.

1. Do you feel satisfied by the service offered to you by the

|  |  | Yes | No |
| --- | --- | --- | --- |
| A | Doctors |  |  |
| B | Nurses |  |  |
| c | Pharmacists |  |  |

1. What line of treatment are you currently in? (1) First (2) Second
2. Your current CD4 counts? .......................Your baseline CD4 count? …………………..

# Questionnaire (Swahili)

**SEHEMU A: TAARIFA BINAFSI**

Weka alama ya tiki katika kiboksi

1. Jinsia

Mume Mke

1. Tarehe/Mwaka wa kuzaliwa ……………………………….
2. Hali ya ndoa
3. Si mwanandoa b) Mwanando c) Metengana/kuachana/Mjane
4. Kiwango cha elimu
5. Hakuna
6. Shule ya msingi
7. Shule ya sekondari
8. Chuo/chuo kikuu
9. Kazi
10. Mkulima
11. Nimeajiriwa
12. Sikuajiriwa
13. Mfanyabiashara
14. Ajira binafsi
15. Makazi

| Moshi | Mjini |  |
| --- | --- | --- |
|  | Vijijini |  |
| Nje ya moshi | Mjini |  |
|  | Vijijini |  |

**SEHEMU B: TATHMINI YA UZINGATIAJI WA DAWA**

1. Ni dozi ngapi umekosa kunywa?

- ***Kama hakuna dozi uliyokosa kunywa andika sifuri katika kiboksi***

***- Swali linauliza kuhusu dozi na si vidonge.Kama ni sehemu tu ya dozi ilimezwa, repoti kuwa dozi haikumezwa.***

| Number ya utambulisho ya dawa | Jina la dawa/kifupisho | Idadi ya dozi ulizoandikiwa kwa siku | Idadi ya dozi ambazo hukunywa siku ya jana | Idadi ya dozi ambazo hukunywa siku ya juzi (siku 2 zilizopita) | Idadi ya dozi ambazo hukunywa siku 3 zilizopita | Idadi ya dozi ambazo hukunywa wiki 2 zilizopita |
| --- | --- | --- | --- | --- | --- | --- |
|  |  |  |  |  |  |  |
|  |  |  |  |  |  |  |
|  |  |  |  |  |  |  |

***Wiki 2 zilizopita zinajumuisha jana, juzi and siku 3 zilizopita***

1. Ni lini mara ya mwisho ulikosa kunywa dawa zako za ARV?

| 0 | Sijakosa kunywa |  |
| --- | --- | --- |
| 1 | Zaidi ya miezi mitatu iliyopita |  |
| 2 | Kati ya mwezi 1-3 iliyopita |  |
| 3 | Wiki 2-4 zilizopita |  |
| 4 | Wiki 1-2 zilizopita |  |
| 5 | Ndani ya wiki 1 iliyopita |  |

1. Katika siku 5 zilizopita ni siku ngapi uliwahi kutokunywa dozi yako/zako za ARV?

| 0 | Hakuna |  |
| --- | --- | --- |
| 1 | Siku 1 |  |
| 2 | Siku 2 |  |
| 4 | Siku 3 |  |
| 4 | Siku 4 |  |
| 5 | Siku 5 |  |

**SECTION C: SABABU ZA KUSHINDWA KUZINGATIA MATIBABU**

1. Sababu mbalimbali zinaweza kuchangia mtu kukosa kunywa dawa. Hapa chini ni miongoni mwa sababu zinazoweza kuchangia kukufanya uwe umekosa kunywa dawa.

Ni mara ngapi sababu ifuatayo imekufaya ukose kunywa dawa zako za ARV?

|  |  | 0.Haijawahi | 1. Mara chache | 2.Mara nyingi |
| --- | --- | --- | --- | --- |
| 1 | Nilitaka kujikinga na madhara ya dawa |  |  |  |
| 2 | Nilijiskia kuwa na afya njema |  |  |  |
| 3 | Nilikunywa kilevi |  |  |  |
| 4 | Sikuweza kupata chakula stahiki |  |  |  |
| 5 | Nilimpatia ndugu/rafiki dawa zangu |  |  |  |
| 6 | Imani ya kidini |  |  |  |
| 7 | Sikuelewa vizuri kuhusu dawa na namna ya kutumia |  |  |  |
| 8 | Nilisafiri mbali na nyumbani |  |  |  |
| 8 | Shida ya usafiri kuelekea kiliniki |  |  |  |
| 10 | Dawa zilipotea |  |  |  |
| 11 | Nilikua na vidonge vingi vya kumeza |  |  |  |
| 12 | Nilipatwa na hali ambayo nilidhani inatokana na hizi dawa |  |  |  |
| 13 | Nilisahau tu |  |  |  |
| 14 | Vidonge viliniishia |  |  |  |
| 15 | Nilichoshwa na kunywa vidonge vingi |  |  |  |
| 16 | Nilitingwa na kazi nyingine za kujitafutia kipato |  |  |  |
| 17 | Matatizo mengine ya kiafya |  |  |  |
| 18 | Hofu ya kunyanyapawa na watu (wasio wa familia) |  |  |  |
| 19 | Hofu ya kunyanyapawa na wana familia ( sikutaka mke/mume ajue) |  |  |  |
| 20 | Dawa ziliharibiwa na maji au joto |  |  |  |
| 21 | Ugonjwa ulinizuia kuja kiliniki kuchukua dawa |  |  |  |
| 22 | Kuamka asubuhi sana kwenda kazini. Hamna muda wa kula kwanza |  |  |  |
| 23 | Siamini kama dawa zinafanya kazi |  |  |  |
| 24 | Matatizo ya kifedha. Sikuwa na nauli ya kuja kiliniki |  |  |  |
| 25 | Kutumia bhangi, madawa, nk |  |  |  |

1. Sababu nyinginezo 25.

26.

27.

1. Je unaridhika na huduma unazopata kutoka kwa

|  |  | Yes | No |
| --- | --- | --- | --- |
| A | Madakitari |  |  |
| B | Manesi |  |  |
| c | Wafamasia |  |  |

1. Uko line ya ngapi ya matibabu? (1) Ya kwanza (2) Ya pili
2. Je CD4 zako kwa sasa ni ngapi? ..................ulianza dawa ukiwa na ngapi? ………..
